# Postural Risks in Dental Practice: An Assessment of Musculoskeletal Health

**DOI:** 10.3390/s24196240

**Published:** 2024-09-26

**Authors:** Alexandra Maria Lazăr (Căteanu), Angela Repanovici, Mihaela Ioana Baritz, Mihaela Monica Scutariu, Anca Ioana Tătaru (Ostafe), Ileana Pantea

**Affiliations:** 1 Design Product and Environment Faculty, Transylvania University of Brasov, 500036 Brasov, Romaniambaritz@unitbv.ro (M.I.B.);; 2 Dental Faculty, “Grigore T. Popa” University of Medicine and Pharmacy, 700115 Iasi, Romania; monascutaru@yahoo.com; 3 Faculty of Medicine, Transylvania University of Brasov, 500036 Brasov, Romania; ileana.pantea@unitbv.ro

**Keywords:** dentists, ergonomics, musculoskeletal health, posture assessment, private practice, RULA, REBA, stomatology, wearable sensors

## Abstract

In recent years, Romania’s stomatology private practice sector has seen substantial growth, with many dentists fully committing to building and expanding their own practices, often funded by their personal income. This study aimed to explore how various postures affect the muscle groups of dentists (380), particularly focusing on identifying positions that may jeopardize their musculoskeletal health. A group of dentists effectively participated in this study (10), adhering to their regular work routines while wearing wearable sensors on their backs to monitor posture and activity. The data gathered from these sensors were analyzed using the RULA (rapid upper-limb assessment) and REBA (rapid entire-body assessment) tools. The findings indicated that the head and shoulder movements during dental procedures involved considerable and repetitive angular shifts, which could strain the neck and back muscles and heighten the risk of musculoskeletal problems. Additionally, the standing postures adopted by the dentists were associated with an increased risk of postural issues and greater overall fatigue. Extended periods of trunk and head tilting were also identified as contributing factors to posture-related challenges.

## 1. Introduction

The use of postural analysis techniques is essential for assessing work activities in multiple fields. In the context of thorough ergonomic evaluations, recognizing the risks of musculoskeletal injuries linked to particular work positions is a key strategy for making posture adjustments and enhancing workspace design.

Despite the perception of dentistry as a predominantly sedentary profession, the prevalence of occupational illnesses, particularly those affecting the musculoskeletal system, is alarmingly high.

Studies conducted among dentists worldwide have highlighted cases of permanent disabilities, early retirement, and musculoskeletal pain (MSP) as significant concerns [1]. For instance, research on Dutch dentists has projected that a substantial portion of them may prematurely end their careers due to loco-motor system diseases [2]. Similarly, investigations on English dental staff have underscored hand problems as the most commonly reported work-related injuries [3].

Noteworthy studies by Hamann, Bylund, and others have further shed light on the prevalence of hand issues among dental professionals. Findings have revealed high rates of hand paresthesia among Swedish female workers and a heightened risk of hand problems among American dentists, particularly women [4].

These insights challenge the perception of dental medicine as a low physically demanding profession akin to office or sales roles, as dentists appear to be more susceptible to musculoskeletal issues compared to doctors or lawyers.

Moreover, emerging evidence suggests that even dental students, despite being relatively new to the field, are not immune to musculoskeletal problems [5,6]. This complex landscape underscores the critical importance of understanding and addressing the musculoskeletal health challenges faced by dental professionals, highlighting the pressing need for proactive measures to safeguard their well-being and longevity in the profession.

Previous in-work posture correction techniques for dentists have several significant limitations, which are detailed below:

Low comfort: Many of the existing devices are uncomfortable for long-term use, which may lead dentists to neglect using them.
Limited adaptability: Most techniques do not adapt well to the different positions and working styles of dentists, which limits their effectiveness.
Complexity and cost: Some solutions are complex and expensive, making them inaccessible to many practitioners.
Lack of real-time feedback: Most devices do not provide immediate feedback for correcting posture, which reduces effectiveness in preventing postural problems.

The new device addresses these limitations as follows:

Increased comfort: We put special emphasis on ergonomic design to ensure the comfort of users for long periods.
Adaptability: Our device is adjustable and can be configured to suit different positions and working styles of dentists.
Accessibility: We have optimized production costs to make the device accessible to as many practitioners as possible.
Real-time feedback: Our device provides immediate feedback, allowing dentists to correct their posture on the spot and thus prevent postural problems.

## 2. Material and Methods: Some Aspects of Ergonomic Techniques Applied in This Field

The posture of dentists is important for their well-being in their activities. REBA (rapid entire-body assessment) is a tool used to evaluate and help find solutions to improve posture in various occupations, including dentistry.

Regular exercise and maintaining proper ergonomics can help prevent work-related musculoskeletal issues. We present some aspects:

- Musculoskeletal disorders (MSDs) are general health problems during work in most areas of industry or service [7]. Therefore, in order to avoid serious health problems, it is necessary over time to assess posture, analyze and evaluate current techniques, and use ergonomic standards [8].
- Repetitive processes and manual handling of various materials and tools in the dental field are the major problems in the daily work of dentists [1]. Therefore, musculoskeletal disorders (MSDs) are related to such repetitive processes of twisting, bending, or lifting objects and working in a deficient posture (upright or even sitting) [9]. Thus, in order to improve the efficiency of the activity of the subjects in the defined sample, their position had to be periodically assessed in order to avoid postural problems and to take corrective measures to reduce and/or eliminate musculoskeletal disorders [10].
- The research techniques proposed over time to estimate the level of discomfort and workload associated with the worker’s adoption of different positions during work can be divided into observation techniques and device-based solutions, respectively. In the case of observation techniques, the angular deviation of body sections from the neutral position is obtained by visual observation.
- However, for instrument-based techniques, continuous position monitoring is carried out by devices connected to the worker. Due to the lack of integration in the work process, low costs, and ease of use, observation techniques are still widely used in industry, medicine, services, etc.
- If the system provides the worker with information about his current ergonomic behavior, then the posture could be changed immediately. Moreover, in the long term, associations between certain postures and their danger could be learned to receive immediate feedback.
- Augmented reality (AR) technology can be used during the execution of various activities. Recent developments in sensor technology offer potential for industrial use on a regular basis, unlike other tracking devices, such as remote cameras or magnetic sensors, which are more efficient in virtual environments [11]. An inertial measurement unit (IMU) is a small, cheap, and low-power device suitable for real-time kinematic monitoring of upper limb, lower, or entire human body movements [12]. When these instruments for ergonomic evaluation were connected with several inertial measurement units (IMUs), it was possible to scan the biomechanical model that was further developed to capture a wide range of movements (sensors for movements, system with video-cam, goniometers, inclinometers, etc.).
- For a unitary and effective assessment of the movements and posture of subjects—doctors—the possibility of using evaluation tools of generally accepted application methods, such as RULA (rapid upper-limb assessment) and REBA (rapid entire-body assessment), has been studied during their work in order to determine the level of risk to which the sample of subjects working in the dental field is exposed.
- RULA (rapid upper-limb assessment), developed by McAtamney and Corlett in 1993, assesses the posture, force, and movement associated with sitting or standing loads. Such tasks include computer, medical (stomatology), manufacturing, or retail activities, and those in which the worker is sitting or standing, without performing short or medium distance movements, but only twists or bends [13]. This tool does not require special equipment to provide a rapid assessment of the postures of the neck, trunk, and upper limbs, together with muscle function and external tasks experienced by the human body. A coding system is used to generate a list of actions indicating the level of intervention needed to reduce the risks of injury due to physical loading on the operator.
- REBA (rapid entire-body assessment) was developed by Hignett S. and McAtamney L. in 2000 to provide a fast and easy postural observational analysis tool for the activities performed by the entire human body (static and dynamic), giving a level of action of musculoskeletal risk. The REBA development aims to divide the human body into segments to be individually encoded with reference to motion plans. This application provides a scoring system for muscle activity caused by static, dynamic, fast-changing, or unstable postures [14,15].
- This analysis reflects the fact that the action of connecting segments of the human body is important in the execution of tasks but cannot always be achieved by the action of the upper limbs alone. The final results also provide a level of future action in view of the indications for urgent intervention, whether in the short or long term.

**Figure 1:**
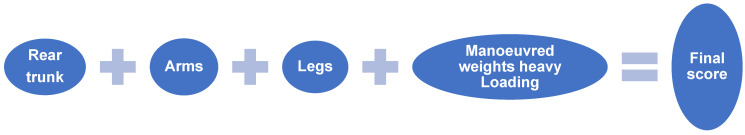
Ergonomic analysis using the RULA application.

For the complete analysis of the posture of subjects—doctors—in the sample, the work schedule in Figure 1 is applied to obtain the final risk score of posture and activity.

The two RULA and REBA applications will provide these scores that can be correlated with each other in order to identify positioning or action deficiencies, both in sitting and standing positions.

In order to analyze the posture and movements of a dentist during a complete medical act, 10 subjects were selected in the primary phase, both male and female, with an average of 42 +/− 0.5 years, with anthropometric dimensions of category 75 percentile, without previously detected musculoskeletal disorders.

The mean duration of a complete medical act per patient was an interval of 55 min (value obtained from the questionnaire preceding this research, based on responses from 380 dentists).

**Figure 2:**
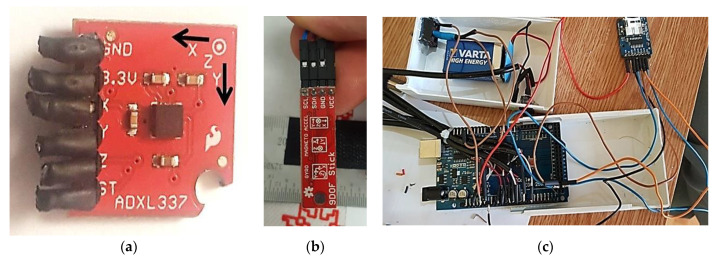
The designed device (**a**–**c**).

Thus, the position of the subjects in the seated position is first analyzed using the device designed attached to their torso (Figure 2), corresponding to the tri-orthogonal Oxyz system, and applying the RULA standard.

The measuring system has the following components:

▪ Acquisition board based on the ATmega 2560 microcontroller;
▪ Measured data recording device on a microSD card;
▪ ADXL 337 type sensors for measuring accelerations on the three axes, x, y, and z, which will be converted into Euler angles using a software application designed for this purpose;
▪ A power source (9 V battery) to eliminate the noise induced by the electrical network in the acquisition process and make the system independent of the computer.

The acquisition board is based on the ATmega 2560 microcontroller, with 16 analog inputs, 54 digital I/O pins, 4 UARTs, a 16 MHz crystal oscillator, a USB connection, a power jack, a set of pins ICSP, and a reset button.

Operating mode: Output signals are analog voltages that are proportional to acceleration. The accelerometer can measure static acceleration due to gravity in tilt sensing applications as well as dynamic acceleration resulting from motion, shock, or vibration. The sensor has a poly-silicon surface with a microstructure built on top of a silicon wafer.

Phase-sensitive demodulation techniques are used, which determine the magnitude and direction of the acceleration. The output of the demodulator is amplified and has a resistance of 32 kΩ as a load.

The measurement system has a software component, a source code that is used by the module to obtain measurement results. To improve the resolution of the measurements, in the initial step of the experiment, a system with 3 inertial sensors with 9 degrees of freedom was designed. Figure 2a–c shows the inertial sensors used. One of the sensors used is the SparkFun 9DOF sensor, which includes an HNC5883L magnetometer, an ITG-3200 gyroscope, and an ADXL345 MEMS accelerometer (b).

The measured data were sent for logging on the I2C interface, and 3 sensors of this type were used, for which 3 development boards with ATmega 328P microcontrollers were used.

The program for data acquisition was adapted to meet the requirements of the experiment. The data were chosen for processing, and the results represented the Euler, yaw, roll, and pitch angles at the output.

On the subject (dentist), the 3 sensors were placed on the head and on each hand. Accelerometer, magnetometer, and gyroscope calibrations were performed for each sensor prior to acquisition.

The acquired data were recorded by the computer, processed, and then visualized using the Processing program.

In order to be able to perform ergonomic analysis, movement and positioning data were taken from the subjects—doctors—during a dental procedure on patients, with comparable duration and energy consumption in both posture situations (sitting and standing).

**Figure 3:**
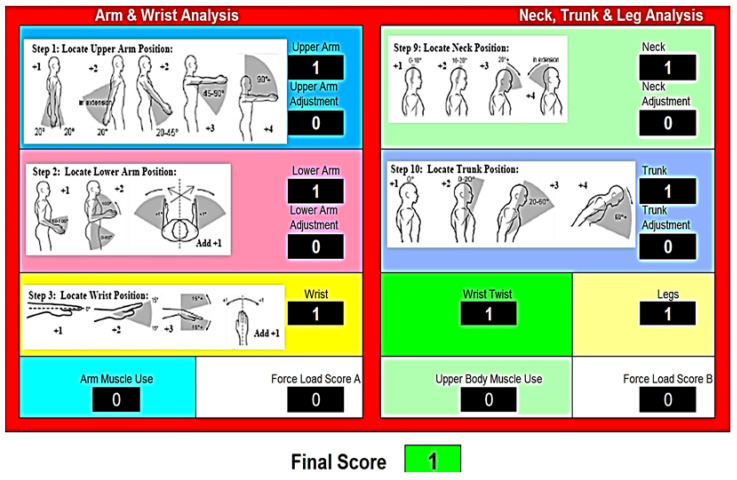
RULA application mean template.

The analysis is based on the use of load coefficients at the torso, head, and upper limb level according to the initial scheme developed in the RULA application (Figure 3).

Each step in the RULA analysis is based on a series of parameters that have been coded by load coefficients and correspond to the data recorded with the device designed and carried out in this research and carried by each of the subjects—physicians—in the sample.

## 3. Results

In this respect, the data recorded by the device (angular values of the torso and head posture) were grouped and mediated over the working hours corresponding to the field-specific medical acts.

Thus, the values recorded by the device represent changes in the position of the head, left shoulder, and right shoulder, respectively (values (x1, y1, z1), (x2, y2, z2), (x3, y3, z3)), according to the example in Table 1, which covers the reference values of the posture, and Table 2, respectively, containing the values corresponding to the time of the medical act.

When using the RULA procedure, the determination of the angles of inclination on the three Oxyz directions is compared with the reference values (when the subject does not perform a medical procedure) with those during a similar medical procedure for all subjects in the same position (sitting or standing).

**Figure 4:**
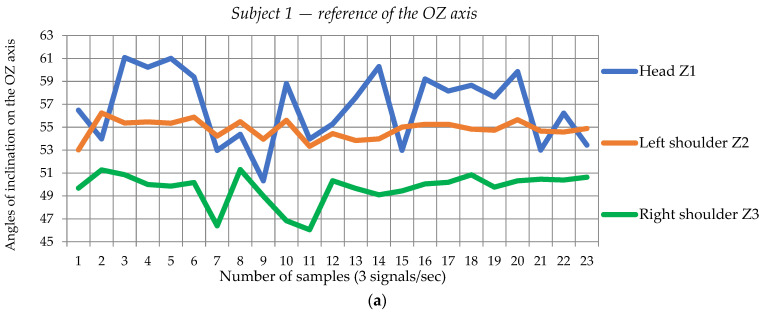

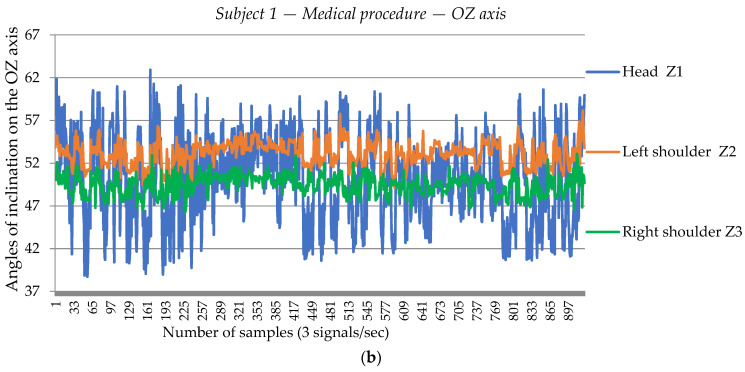
Example of inclination angles for Subject 1 ((**a**)—reference and (**b**)—medical procedure).

Similar values are recorded for each subject that is part of the sample, whether seated for the use of RULA or standing for the use of the complex REBA form. Thus, from the graphs shown in Figure 4, one can see and then determine the differences in postural inclination that constitute input data for the application of the RULA procedure. For this example—Subject 1, sitting position in the medical procedure—the mean postural tilt values of the head in the direction of the OZ axis is 50.2°, the left shoulder of 53.14°, and the right shoulder of 49.5° relative to the reference values for head 59.31°, left shoulder 57.31° and right shoulder 51.93°. Therefore, Subject 1, in the working position, has a rigid, tense state, positioning the head and the two shoulders with values very different from angles of inclination. Although the two shoulders exhibit similar variations in tilt values (minimum 47°, maximum 57°), the inclination of the head, after the same Oz axis, is highly variable, having limits between the minimum value of 38° and the maximum of 62.5°. These tilt oscillations indicate a frequent tilt movement of the front–back head, which can lead to maximum stress on the joints and head–neck muscles. From the similar analysis carried out in the other two directions (Ox and Oy) of the signals captured with the system designed and carried out in this research, it can be concluded that the movement of the head, left shoulder, and right shoulder, in the case of subjects—doctors—in the working procedure involves large, repetitive angular displacements, which can cause tension of the neck and back muscles and develop musculoskeletal problems or even increase the risk of additional fatigue.

From the similar analysis carried out in the other two directions (Ox and Oy) of the signals captured with the system designed and carried out in this research, it can be concluded that the movement of the head, left shoulder, and right shoulder, in the case of subjects—doctors—in the working procedure involves large, repetitive angular displacements, which can cause tension of the neck and back muscles and develop musculoskeletal problems or even increase the risk of additional fatigue.

These observations are supported by the results of the application of the RULA procedure, as set out in Table 3 for the subjects working in the sitting position.

The average angular positioning values on the three axes of the head and shoulder of subjects—physicians—working in the sitting position are found by applying the RULA procedure Ver. 2. The final score for the four subjects (in sitting position) exceeds the accepted limit and, therefore, requires specific evaluations to identify the constructive aspects leading to the improvement in the posture of the investigated subjects.

For the situation where the investigated subjects worked in the standing position, the values acquired with the device designed and used are summarized in Table 4. In this case, the REBA procedure was used, which also takes into account the effects of lower limb posture on the determination of the level of risk.

These aspects are highlighted by the determination of intermediate coefficients in order to identify future intervention areas for improvement in subjects’ posture. The final report of the REBA procedure is much more complex and highlights all the possibilities of positioning the human body in a standing activity but without handling heavy weights. Since the device designed and released in this research is carried by physicians also in the upper part of the body, then the angular values measured refer to the trunk, head, and upper limbs, and for the lower limbs, direct observations of their behavior.

Through the use of these applications, RULA and REBA were able to highlight the stages of these positions during a medical act in the dental office when the subject (dentist) works in a sitting or standing position. An interesting case is that of the dental doctor, who works combined, using both the sitting and standing position for different durations each. These subjects (No. 5 and No. 9) from the sample were removed from the general analysis and were considered in another study in the same field by the authors of this paper.

From the evaluation of the RULA and REBA coefficients determined for the analyzed position, it is found that, in both situations, it is the same for the subjects with the same position, values (RULA = 7 and REBA = 11), which warns of the risk level of installing musculoskeletal disorders. The angles determined by these methods are compared with the angles obtained from the device designed to equate the results.

## 4. Discussion

The authors have meticulously reviewed existing literature pertaining to the identified issue and have drawn insightful conclusions from both the synthesized research findings and direct observations of dental specialists in neighboring dental practices. This comprehensive examination culminated in the inception of an experimental study aimed at investigating these critical aspects.

Upon the implementation of the RULA procedures, tailored to analyze the upper body’s behavior, and the REBA methodology, specialized in evaluating the entire human body’s actions, several pivotal findings have emerged:

− Prolonged maintenance of a trunk tilt coupled with an additional head tilt, characterized by twisting and bending motions, has been associated with an increased risk of developing posture-related issues among the doctor subjects, as well as potential musculoskeletal problems in the upper body.
− The standing posture adopted by physician subjects has been linked to heightened risks of postural challenges and may contribute to elevated levels of overall bodily fatigue.

The utilization of this portable device enables swift identification of the specific body regions subjected to such exertions, facilitates risk level determination, and facilitates the formulation of tailored solutions for individual cases. Its ease of attachment, positioning, and rapid data recording render this device a highly advantageous system compared to more intricate alternatives like video and electromagnetic systems, thereby promoting its widespread applicability.

The data acquired through this innovative device underscore the pronounced susceptibility of dentists to musculoskeletal pain (MSP). Further investigations are warranted to delve into the nuances of body posture and physical assessments of dentists in correlation with self-reported musculoskeletal pain [15,16]. Additionally, the authors advocate for the incorporation of ergonomic training for dentists as a pivotal measure to mitigate musculoskeletal disorders (MSDs). While the current device primarily focuses on muscular stress in the upper body, the authors propose the development of an analytical tool for the lower body and emphasize the necessity for subsequent testing to validate the accuracy of the resultant data [17,18,19].

## 5. Conclusions

This research highlights the critical importance of understanding the impact of posture on the musculoskeletal health of dentists, a profession often characterized by prolonged and repetitive movements. By identifying specific postural risks, this study provides valuable insights that can lead to the development of targeted interventions aimed at reducing the incidence of musculoskeletal disorders among dental practitioners.

Furthermore, the findings underscore the necessity for ergonomic training and the implementation of best practices in workspace design, which can significantly enhance the well-being and productivity of dental professionals. Ultimately, this research not only contributes to the body of knowledge in occupational health but also serves as a foundation for future studies aimed at improving the working conditions and overall health of those in the dental field. By prioritizing the well-being of dentists, we can ensure better patient care and a more sustainable practice environment [20,21,22].

## Figures and Tables

**Table 1:** Reference values of tilt angles for the sitting position of the subject—doctor—measured by the proposed device (illustrated for Subject No. 1).

Head			Left Shoulder			Right Shoulder		
X1	Y1	Z1	X2	Y2	Z2	X3	Y3	Z3
74.66	67.51	56.48	75.76	71.37	53.02	75.13	72.62	49.68
90.91	91.26	53.98	77.89	72.2	56.24	81.41	79.33	51.27
86.29	83.3	61.08	78.47	73.54	55.37	79.23	76.85	50.85
84.44	80.34	60.24	77.47	72.11	55.46	76.88	74.48	50
86.42	83.56	61.01	77.39	72.06	55.35	77	74.68	49.86
84.7	81.1	59.38	77.54	71.95	55.87	77.29	74.87	50.17
87.92	87.24	52.97	76.98	72.21	54.23	77.5	76.9	46.39
……	……	……	……	……	……	……	……	……

**Table 2:** Values of the angles of inclination corresponding to the performance of the medical act for the sitting position of the subject—doctor—measured with the proposed device (illustrated for Subject No. 1).

Head			Left Shoulder			Right Shoulder		
X1	Y1	Z1	X2	Y2	Z2	X3	Y3	Z3
75.89	65.54	61.07	76.32	71.57	53.85	77.12	74.51	50.49
91.07	91.7	57.71	77.2	71.89	55.21	76.1	73.53	50.07
81.67	74.69	61.87	77.34	72.07	55.23	77.98	75.4	50.74
91.85	92.58	54.25	77.61	72.45	55.21	77.82	74.5	52.11
80.2	74.77	57.59	77.69	73.16	54.22	75.77	72.98	50.37
79.62	73.02	59.04	76.28	71.32	54.39	76.13	73.68	49.87
……	……	……	……	……	……	……	……	……

**Table 3:** RULA score for sitting position in the working procedure.

Posture		Mean Ox Axis Tilt	Average Tilt on the Oy Axis	Mean Inclination on the Oz Axis	Mean Resulting Tilt	RULA Score	Comments
Sitting position	Subject 1	76.8	73.8	51	118.12	7	Immediate investigation and modifications
	Subject 2	77.64	74.5	51.42	119.33	7	Immediate investigation and modifications
	Subject 3	77.9	74.8	52.5	120.02	7	Immediate investigation and modifications
	Subject 7	80.81	77.52	54.87	124.71	7	Immediate investigation and modifications

**Table 4:** REBA score for standing position in the working procedure.

Posture		Mean Ox Axis Tilt	Average Tilt on the Oy Axis	Mean Inclination on the Oz axis	Mean Resulting Tilt	REBA Score	Comments
Standing position	Subject 4	79.5	71.23	54.03	119.63	11	Very high risk, immediate action needed
	Subject 6	77.44	74.14	52.35	119.31	11	Very high risk, immediate action needed
	Subject 8	80.26	77.02	53.54	123.46	11	Very high risk, immediate action needed
	Subject 10	80.01	76.86	52.66	122.81	11	Very high risk, immediate action needed

## Data Availability

The original contributions presented in the study are included in the article, further inquiries can be directed to the corresponding author.
